# Bacteriophage therapy beyond antibiotics: emerging innovations for infectious and non-infectious diseases

**DOI:** 10.3389/fcimb.2026.1879718

**Published:** 2026-07-08

**Authors:** Biyi Zhang, Xutong Shu, Abdullah Ibna Masud, Joyoshrie Karmakar, Jie Fan, Ishatur Nime, Mrityunjoy Acharjee, Fan Pan, Md. Sharifull Islam

**Affiliations:** 1 Faculty of Pharmaceutical Sciences, Shenzhen University of Advanced Technology (SUAT), No. 1 Gongchang Road, Guangming District, Shenzhen, China; 2 Center for Cancer Immunology, Institute of Biomedicine and Biotechnology, Shenzhen Institutes of Advanced Technology (SIAT), Chinese Academy of Sciences (CAS), 1068 Xueyuan Avenue, Shenzhen, China; 3 Department of Microbiology, Stamford University Bangladesh, Dhaka, Bangladesh; 4 College of Clinical Medicine, The First Affiliated Hospital, Henan University of Science and Technology, Luoyang, China; 5 Key Laboratory of Environment Correlative Dietology, College of Food Science and Technology, Huazhong Agricultural University, Wuhan, Hubei, China

**Keywords:** antimicrobial resistance, bacteriophage therapy, biofilm eradication, personalized medicine, phage antibiotic synergy

## Abstract

The advancement of synthetic biology and the rise of antimicrobial resistance have led to the development of bacteriophage therapy for more than antibacterial applications. This review focuses on applications to multidrug-resistant infections, biofilm diseases, cancer research, veterinary medicine and animal production. Recent research suggests phages can be used in combination with antibiotics to enhance treatment of large multidrug resistant pathogens such as *Pseudomonas aeruginosa*, *Acinetobacter baumannii* and *Klebsiella pneumoniae*. This could also help to restore antibiotic sensitivity by making bacteria change resistance related structures or mechanisms. Despite this, there are several challenges for the use of phage therapy prior to its widespread clinical application, including phage resistance, difference in patient response, unknown pharmacokinetic parameters, immune issues, and unclear regulatory guidelines. Additionally, in some cases, phages could also play a role in horizontal gene transfer, raising further safety concerns. Beyond antimicrobial therapy, phage display platforms derived from M13, T7 and λ phages have enabled the identification of tumor-targeting peptides, the development of immunomodulatory constructs, and targeted delivery of therapeutic molecules. Over 100 clinical cases and 44 registered trials support the generally favorable safety profile of personalized phage therapy, and highlight the need for better treatment standardization, controlled clinical evaluation, and better regulatory processes. Additionally, engineered phages expressing biofilm degrading enzymes represent promising tools for disrupting matrix-embedded bacterial communities associated with chronic infections and medical devices. In summary, CRISPR-based engineering and genome refactoring highlight the potential of phage-based therapeutics as complements to conventional antimicrobial therapy, although their broader use depends on overcoming biological, clinical, and regulatory challenges.

## 1 Introduction

Bacteriophage (phage) therapy has reemerged as a promising strategy to address the escalating global threat of antimicrobial resistance (AMR). As the effectiveness of conventional antibiotics continues to decline, phages viruses that specifically infect bacteria are attracting renewed attention as targeted antibacterial agents. Despite their therapeutic potential, clinical application of phage therapy often remains highly personalized because most bacteriophages exhibit a narrow host range, infecting only specific bacterial strains. In principle, phage cocktails designed to target multiple bacterial receptors could broaden therapeutic coverage. However, the immense diversity of bacterial species and the complex ecological and evolutionary interactions among phages present significant challenges for the rational design of broadly effective phage formulations (Kim et al., 2024). Nevertheless, growing experimental and clinical evidence demonstrates that bacteriophage therapy can effectively eliminate bacterial pathogens that exhibit resistance to multiple classes of antibiotics.

Beyond direct bacterial killing, phages can impose evolutionary pressures that reshape bacterial physiology in therapeutically beneficial ways. Certain lytic phages select against bacterial traits associated with virulence or antibiotic resistance. For instance, phages that utilize antibiotic efflux pumps as receptors can select for bacterial mutants that evade phage adsorption by downregulating or disrupting these pumps. As a consequence, such mutants frequently display impaired efflux activity and increased susceptibility to antibiotic treatment (Chan et al., 2016). Similarly, phages that recognize structural virulence determinants, including capsular polysaccharides, can drive the emergence of capsule deficient bacterial variants that exhibit reduced virulence while acquiring resistance to the infecting phage (Smith and Huggins, 1982). These evolutionary tradeoffs highlight the potential of phage therapy not only to eliminate pathogens but also to modulate bacterial phenotypes in ways that enhance the efficacy of conventional antimicrobials.

Evidence from animal infection models further supports the therapeutic potential of bacteriophages against multidrug-resistant pathogens. In murine models of intraperitoneal *Pseudomonas aeruginosa* infection, concurrent administration of bacteriophages has achieved survival rates approaching 92% (Watanabe et al., 2007). Comparable results have been reported in mouse models of *Staphylococcus aureus* abscess formation, where phage treatment significantly reduced bacterial load and prevented abscess development when administered simultaneously with bacterial challenge. Moreover, delayed phage administration has also demonstrated therapeutic efficacy: a single phage dose delivered four days after infection reduced bacterial counts by approximately two orders of magnitude, whereas repeated dosing achieved reductions of nearly four orders of magnitude (Capparelli et al., 2007). These findings highlight the capacity of bacteriophages to control established infections and support their potential translation into clinical practice.

Though there is great promise with phage therapy, there are several biological and systemic challenges. One major biological challenge is the host range of most bacteriophages, which is limited, meaning that they don’t disrupt a healthy microbiota, but they may require complex ‘phage cocktails’ to exert a broad therapeutic effect against various bacterial strains (Łobocka et al., 2021; Petrovic Fabijan et al., 2023). Additionally, bacteria have developed elaborate anti-phage protective mechanisms such as Restriction-Modification systems and CRISPR (cassette of CRISPR-interacting RNA) adaptive immune systems, which can cause swift resistance and treatment failure (Egido et al., 2022). Regulatory hurdles are still big problems from a clinical point of view. Current regulatory frameworks in many parts of the western world are targeted at static chemical drugs and poorly suited for ‘living’ therapeutics, which evolve and need to be tailored to the individual infection (Petrenko and Gillespie, 2017; Pires et al., 2020).

The concept of phage therapy dates back more than a century. In 1919, Félix d’Herelle first reported the successful therapeutic use of bacteriophages to treat infants suffering from bacterial diarrhea. During the 1930s, phage therapy was widely applied to treat bacterial infections in humans and animals, preceding the large-scale commercialization of penicillin (Summers, 2012). Subsequently, dedicated phage therapy research centers were established in Tbilisi, Georgia, and Wrocław, Poland, both of which remain active today. However, interest in phage therapy declined sharply in Western countries following the introduction of broad-spectrum antibiotics in the 1940s.

Antibiotics were widely perceived as more convenient therapeutic agents because of their broad activity against diverse bacterial pathogens, whereas phage therapy requires precise matching between phage and bacterial host. In addition, geopolitical tensions during the post–Second World War period contributed to skepticism toward therapeutic approaches developed within the former Soviet Bloc, further limiting the global adoption of phage therapy for several decades (Summers, 2012).

In addition to being natural bacteria predators, bacteriophages can be used in modern medicine. Their nanoscale size, the ability to target specific locations, and their compatibility with genetic engineering have made them promising biological nanotechnology tools for applications beyond antimicrobial resistance (Petrenko and Gillespie, 2017; Mutalik et al., 2026). The researchers can tap into the tumor microenvironment (Islam et al., 2023a) by using phages as ‘killers’ of oncogenic bacteria and as programmable scaffolds for drug delivery and imaging. As a result, a ‘dual-action’ approach is emerging, in which phages not only target the underlying microbial drivers of disease, but also directly deliver anticancer payloads (Mutalik et al., 2026).

In recent years, advances in molecular biology, synthetic biology, and nanobiotechnology have revitalized interest in bacteriophages as multifunctional biomedical tools. Contemporary cancer treatment primarily relies on surgery, chemotherapy, radiotherapy, and hormone-based interventions (Strathdee et al., 2023). Alongside these conventional approaches, emerging strategies such as immunotherapy including monoclonal antibodies, cytokine-based immune stimulation, and dendritic-cell-based therapies have demonstrated substantial therapeutic promise (Manivannan et al., 2022). Within this rapidly evolving landscape, phage-based technologies have gained prominence as versatile platforms for targeted molecular discovery and therapeutic delivery.

Phage display technology, particularly using filamentous M13 bacteriophage, has become a powerful tool for identifying peptides and antibodies with high specificity for tumor-associated targets. Since its optimization for human antibody generation during the late 1980s and early 1990s, phage display has enabled the discovery of tumor-homing peptides that can be developed for targeted diagnostics and therapeutics (Ragothaman and Yoo, 2023). In addition, phage-derived platforms such as phage DNA vaccines and phage display–based vaccines are being explored as innovative strategies for vaccine development and cancer immunotherapy (Manivannan et al., 2022). Together, these advances underscore the expanding role of bacteriophages as versatile tools in modern biomedical research and therapeutic innovation.

## 2 Phage against antibiotic resistance

There is a large amount of preclinical data and more recent evaluation of phage therapy in clinical settings that has proved this as a potent approach to the elimination of MDR pathogens that are resistant to traditional antibiotics (**Table 1**) (Petrovic Fabijan et al., 2023; Pal et al., 2024). Therapeutic success in systemic infection models is mainly determined by the critical dose and timing of the phage. For instance, for vancomycin-resistant *Enterococcus faecium* and *E. faecalis*, there has been a clear dose-dependent rescue effect, namely, increasing the multiplicity of infection significantly lowered bacterial burden in the blood and increased host survival rate (Biswas et al., 2002; Cheng et al., 2017). *Vibrio vulnificus* sepsis, on the other hand, reveals a tightly coupled therapeutic window for effective phage intervention which can only be achieved when treatments are administered simultaneously or following bacterial challenge (Cerveny et al., 2002). The results highlight the importance of accuracy for dosing and administration of the drugs to achieve clinical translation for each pathogen’s pathophysiology. In addition to direct bactericidal activity, the relationship between phages and horizontal gene transfer is complicated for use in the clinic. The recent discovery of ‘paradoxical dissemination’ of antibiotic resistance genes by phage predation points to the need for more research in the field. The activity of phages decreases the overall microbial biomass, but at the same time they lower the speed at which individual bacterial species become spatially separated when an expanding colony is formed. This also raises the “intermixing index” between strains, and this provides greater chance for transmission of the conjugative transfer of resistance plasmids, including R388, even if those plasmids have a detrimental effect on the host (Ruan et al., 2024). The clinical consequences of this have a major impact. In polymicrobial systems with environments, such as the human gut microbiome and chronic burn wounds, the process of incomplete clearing by phages may unwittingly promote the propagation of resistance plasmids within “persister” cells and commensals living in the environment. To reduce this risk, it is imperative that future therapeutic strategies become “evolution-informed” and incorporate plasmid-dependent phages (Smith et al., 2023). The specialized viruses specifically target the conjugation machinery (e.g., pili) of resistant bacteria and provide a selective pressure that actively punishes carriage of resistance plasmids (Harrison, 2015). Clinicians will be able to use the standard lytic phages with plasmid-dependent phages to kill pathogens and to simultaneously reduce the local reservoir of antibiotic resistance in the patient’s microbiome. These results underscore the importance of using phages for the treatment of AMR infections, but recent developments have extended the possibilities of phages to other areas of clinical and non-infectious use (Islam et al., 2019).

**Table 1:** Use of phage against antibiotic resistance pathogen.

Category	Animal/Food	Purpose	Pathogen	Reported efficacy	Citation
Pre harvest	Chickens	To reduce colonization	Campylobacter jejuni/coli	≥1–2 log reductions	(Olson et al., 2021)
	Food animals	To reduce enteric pathogens shedding	E. coli O157:H7, Salmonella spp.	Oral or rectal phage reduced fecal shedding.	(Moye et al., 2018)
	Poultry	To reduce Salmonella on raw poultry pieces	Salmonella enterica	Significant decreases compared with controls.	(Sukumaran et al., 2016; Islam et al., 2020)
Across pre/post harvest	Across pre/post-harvest & foods	Phage in food production & processing.	Various (Salmonella, Listeria, E. coli, Campylobacter, Vibrio)	Reduction of target pathogen loads (often by 1–3 logs).	(Bumunang et al., 2023)
Post harvest	Fresh fruits	To Evaluate Listex™ P100	Listeria monocytogenes	Significant reductions on fruit surfaces.	(Oliveira et al., 2014)
	Cooked turkey & roast beef.	To Test LISTEX P100	Listeria monocytogenes	Improved reduction in some matrices.	Chibeu, Agius (Chibeu et al., 2013)
	Dry-cured ham & surfaces	To Apply P100	Listeria monocytogenes	Listericidal effects on inoculated ham slices and reduced biofilms on equipment surface	(Iacumin et al., 2016)
	Ham	To Evaluate ListShield™, Listex™ P100	Listeria monocytogenes	Reduce L. monocytogenes on foods and surfaces;	(Gutiérrez et al., 2017)
Animal	Shrimp	To Evaluate phage cocktails.	Vibrio spp., Aeromonas spp.	Reduced Vibrio/Aeromonas counts	(Piranaghl et al., 2023)
Commercial	Carcasses and meat	To Evaluate EcoShield™	E. coli O157:H7	Reductions of E. coli O157 on treated carcasses/meat.	(Vikram et al., 2021)
Human trial	Children	To Test oral coliphage.	Diarrheagenic E. coli (coliphage target)	Did not demonstrate convincing therapeutic benefit for the coliphage	(Sarker et al., 2016)
	Healthy volunteers	To Recovery of orally given coliphage T4	E. coli (coliphage study)	Fecal coliphage detected transiently and no major adverse events reported.	(Bruttin and Brüssow, 2005)
	Topical wound RCT	Phage cocktail vs standard care in burn wound infections.	Pseudomonas aeruginosa (often MDR)	The phage arm had mixed efficacy compared with standard of care.	(Jault et al., 2019)

## 3 Clinical applications of phage therapy

The use of phages has developed from simple preparations to a high precision pharmaceutical pipeline since the time of Félix d’Hérelle’s first use of phages in the treatment of bacterial dysentery in 1919. The clinical interest in the specificity of phages has been renewed in the mid-20th century, when broad-spectrum antibiotics dominated as the primary therapy, but with the emergence of multidrug-resistant pathogens, phages are being focused on as a therapy that targets unique surface receptors found in bacteria but not in host microbes (Petrovic Fabijan et al., 2023; Kim et al., 2025).

One of the most important recent developments in the clinical use of phages has been the worldwide trend of standardization of diagnostics. In 2024, groups such as the European Committee on Antimicrobial Susceptibility Testing started to formalize Phage Susceptibility Testing protocols to make sure that clinical assays are reproducible and predictive of *in vivo* results (Kiljunen and Resch, 2024; Gonzalez Moreno et al., 2025). The change reflects the lack of standardization of assays in the past, which previously prevented widespread clinical validation. In addition, the establishment of “magistral” regulatory structures in Belgium and Portugal marks a paradigm shift, where hospitals may employ laboratory-produced phage products that have not been approved by any authority for use in the patient population who are unable to receive any other treatment (Kim et al., 2025). Artificial intelligence and synthetic biology (Zhou et al., 2026) help accelerate the start of the clinical modernization of phage therapy. In recent research, the use of machine learning models to quickly predict phage-host compatibility and the engineering of phages with broad host range to preemptively overcome bacterial resistance are mentioned (Zhou et al., 2026). With the use of centralized banks of phages, like those developed in the United States and Europe, clinicians can now aspire to a more scalable ‘off-the-shelf’ approach while still enjoying personalized medicine precision (Sawa et al., 2024).

## 4 Phage design and clinical trials

There are essentially two main strategies for engineering phages to achieve specific functions. One approach is to modify the genome of an existing, well-characterized phage to give it new or improved properties. The other is a build-by-design strategy, in which synthetic genomics is used to assemble phages based on established principles of phage biology (Pires et al., 2016). Although phage synthetic genomics is still at an early stage, it offers substantial potential because it is not restricted by the limitations of naturally occurring phages. In contrast, tools and methods for direct phage genome modification are more mature, and several engineered phages have already been used in therapeutic settings (Nick et al., 2022).

Modern phage engineering is a collection of advanced strategies to deal with the constraints of natural isolates. This is an important development, because the limited host range of natural phages is a major obstacle to translation for clinical use. Advanced high throughput methods allow for identification of specific genes that encode bacteriophage receptor recognition, on which specific changes can be made or in-host recombination or out-of-host synthesis can be used to swap genes. It makes it possible to strategically change the host range, allowing phages to attack a wide variety of bacterial strains or to develop resistance mutations during treatment (Jia et al., 2023; Peng et al., 2024).

A specific tool for broadening host-range and specificity is the engineering of receptor binding proteins. The knowledge of the interactions between the phage receptor recognition proteins and host receptors can be used as a guide to modify or replace them, to change the receptor range of the bacteriophage. The yeast-based phage switching platforms have been further developed to generate more predictable and longer-range host of phage tail protein, which effectively overcome the variation of bacterial surface receptor (Pirnay, 2020). Infections by resistant pathogens have been efficiently avoided with these structure-guided design strategies. The delivery system of CRISPR-Cas has become an advancing approach for precision antimicrobial therapy. Phages can be modified to carry the CRISPR-Cas system, a tool for killing bacterial cells based on their sequence-specific properties, which avoids the use of the classical lytic pathway. Moreover, phages can be engineered as adjuvants to antibiotics and carry CRISPR-Cas systems that can restore antibiotic sensitivity to multidrug-resistant bacteria, thus driving the drug resistance (Pires et al., 2020). The incorporation of CRISPR-Cas systems allows for more precise genetic engineering, potentially making phages carriers for functional genes/proteins that will improve therapeutic results (Jo et al., 2023). One of the challenges that depolymerase engineering is attempting to tackle is that of infections associated with biofilms which are notoriously difficult to treat with traditional antibiotics. Phages can be genetically modified to express or amplify depolymerase enzymes capable of degrading the EPS matrix of biofilms, thus allowing the embedded bacteria to be targeted by the phages and to become susceptible to antibiotics. This is especially important for decontamination of infected implanted medical devices where the formation of biofilms makes sterilization and treatment difficult. Phages, which are engineered to carry inbuilt properties like biofilm-degrading enzyme constitute a major step forward in the treatment of chronic device associated infections (Barbu et al., 2016).

However, insufficient attention to fundamental pre-clinical and pharmacologic considerations has contributed to several well-publicized setbacks in early clinical trials of phage therapy (Sarker et al., 2016; Jault et al., 2019). In recent years, the number of phages based clinical trials listed on ClinicalTrials.gov has risen markedly. Of the 44 interventional trials with therapeutic goals, 29 have been registered since the start of 2020. Most of these studies plan to use phages isolated from the environment, but three trials involve CRISPR-enhanced phage products. While many protocols focus on the direct bactericidal activity of lytic phages, a growing subset aims to exploit phages’ capacity to disrupt biofilms, particularly those that complicate decontamination or sterilization of infected implanted medical devices (Islam et al., 2021).

## 5 Medical implications

Beyond their use in treating acute bacterial infections, phages may also have value in managing chronic diseases in which bacteria contribute to disease mechanisms, as summarized in **Table 2**. For instance, the gut liver microbiome axis has been linked to inflammatory responses in alcoholic liver disease, non-alcoholic fatty liver disease (NAFLD), and non-alcoholic steatohepatitis (NASH), as well as in irritable bowel syndrome (IBS). Although the exact causal pathways are not yet fully defined, pre-clinical data are encouraging (Duan et al., 2019; Yuan et al., 2019). Clinical trials are being planned to test whether phage therapy can selectively target *Enterococcus faecalis* and *Klebsiella pneumoniae* in the gut microbiome, thereby slowing progression to liver disease, and to reduce invasive *Escherichia coli* linked to Crohn’s disease. Recent work has also identified a prophage active against *Helicobacter pylori*, raising the possibility that phage-based approaches could be used to address the etiologic role of this organism in gastric ulcer disease and gastric cancer (Ferreira et al., 2022; Islam et al., 2023a). In addition, phage prophylaxis might be used to limit transmission of pulmonary pathogens such as *Mycobacterium tuberculosis* (Hatfull, 2014). Filamentous phages offer another avenue for innovation: their structural properties allow them to be incorporated into hydrogels, which could be applied to reduce or prevent biofilm formation on implanted medical hardware, such as prosthetic devices (Jackson et al., 2021). A further potential application is deliberate “grooming” of the gut microbiome by inducing prophage activation with specific medications or dietary components, including Stevia rebaudiana and bee propolis extracts (Boling et al., 2020; Hu et al., 2021; Sutcliffe et al., 2021). However, these approaches also raise important safety considerations. The use of broad-spectrum or targeted phages may unintentionally alter the composition of the native microbiota, potentially leading to dysbiosis and disruptions in microbial ecosystem stability. Moreover, phage–bacteria co-evolution within the gut may expand the host range of lytic phages, increasing the possibility of affecting beneficial commensal bacteria. Another concern is prophage activation, which can be triggered by factors such as antibiotics, dietary components, or inflammation. This process may facilitate horizontal gene transfer and lysogenic conversion, contributing to the spread of virulence factors and antimicrobial resistance genes. Therefore, careful evaluation of the long-term safety and ecological consequences of these strategies will be essential before their widespread clinical application.

**Table 2:** Use of phage as a treatment strategy.

Pathogen/disease	Result	Phage used	Success/failure	Efficiency	Citation
Difficult-to-treat infections (100 cases)	Majority showed clinical benefit	Personalized phage cocktails	Success	Positive outcomes in majority	(Pirnay et al., 2024)
MDR *Acinetobacter baumannii*	Improvement in clinical case	Custom phages	Success	Pathogen clearance	(Qu et al., 2024)
MDR *Escherichia coli* (UTI)	Novel lytic phage with strong killing	vB_Ec_ZCEC14	Success	Strong *in vitro* activity	(Ismael et al., 2024)
*Pseudomonas aeruginosa* (implant infection, cat)	Cleared infection with phage + antibiotic	Personalized Pseudomonas phages	Success	Resolution in reported case	(Braunstein et al., 2024)
*Klebsiella pneumoniae* (case)	Clinical benefit, reduced virulence with phage resistance	Personalized K. pneumoniae phages	Success	Clinical improvement	(Li et al., 2023)
*Acinetobacter baumannii*	Synergistic killing with antibiotics	ΦFG02 and others	Success	Significant synergy	(Orozco-Ochoa et al., 2025)
Ventilator-associated pneumonia	Improved outcomes in experimental model	Specific phages	Success	Improved survival/clearance	(Weissfuss et al., 2025)
*Acinetobacter baumannii* (KL types)	Tailored cocktails targeted most isolates	Optimized cocktail	Success	89.1% isolates targeted	(Liu et al., 2025)
*Staphylococcus aureus* (biofilm/DFI)	Disrupted biofilms, case evidence	Anti-staphylococcal phages	Success/mixed	Biofilm reduction	(Liu et al., 2024)
*Pseudomonas aeruginosa*	Synergy with antibiotics	Pseudomonas phages	Success	Enhanced killing	(Fatima and Hynes, 2025)
*Klebsiella pneumoniae*	New lytic phages with potential	New lytic phages	Promising	Effective *in vitro*	(Le Bris et al., 2025)
MDR pathogens (clinical trials)	Mixed results; personalized better	Cocktails/personalized phages	Mixed	Variable outcomes	(Uchechukwu and Shonekan, 2024)
*Escherichia coli*	Engineered phages with CRISPR payloads	Engineered phages	Success	Broad killing range	(Gencay et al., 2024)
*Acinetobacter baumannii*	Evidence base growing	Multiple phages	Promising	Biofilm eradication, synergy	(Tan et al., 2023)
Severe bacterial infections in ICU	Safe and often beneficial	Customized phages	Success	Clinical/microbiological improvement	(Sawa et al., 2024)
*Escherichia coli*	Novel phages active against MDR isolates	New lytic E. coli phages	Success	Significant lytic activity	(Islam et al., 2024)
*Staphylococcus aureus*	Biofilm reduction	Anti-staph phages	Success	Substantial reduction	(Mobarezi et al., 2025)
*Klebsiella pneumoniae*	Phage PK2420 reduced infection	PK2420	Success	Reduced bacterial burden	(Chen et al., 2025a)
*Acinetobacter baumannii*	Broad-spectrum phage P425 active	P425	Success	Active against 9 isolates	(Lin et al., 2025)
Phage + antibiotic (review)	Enhanced killing, reduced resistance	Multiple phages	Success	Synergy metrics positive	(Anastassopoulou et al., 2024)

Among the available phages and phage-based vectors, a major advantage of phage M13 is that it can be produced and purified with relatively little effort compared with many other systems. The bacterial host essentially serves as a continuous factory for M13 production (Rakonjac, 2012; Rami et al., 2017). However, M13 phage display also has several drawbacks. These include challenges in constructing high-quality cDNA libraries, constraints imposed by the requirement to generate fusion proteins between the phage coat protein and heterologous peptides, and potential issues related to secretion of phage particles into the periplasmic space. In contrast to M13, which carries a single-stranded DNA genome, T7 phages possess double-stranded DNA. This feature makes T7 less susceptible to replication-associated mutations and generally more genetically stable. In addition, T7 is not dependent on the host protein secretion machinery, which can simplify display of foreign peptides. Lambda phage has a linear double-stranded DNA genome that circularizes after infection of Escherichia coli, allowing replication to proceed. A key limitation of lambda as a cloning or display vector is its relatively modest packaging capacity, accommodating only about 35–50 kb of foreign DNA (Savageau, 2013). Lambda phage adsorbs to *E. coli* via the maltose transport system; consequently, efficient infection requires that the culture medium contain maltose (Pirnay, 2020; Jo et al., 2023). After the phage DNA enters the cell, the cohesive 12-bp “sticky” ends at both termini of the genome anneal, forming a circular molecule. Approximately 45 minutes after infection, bacterial lysis occurs, newly assembled phages are released, and the infection spreads to neighboring cells, repeating this cycle (Rakonjac, 2012; Dennehy and Abedon, 2021).

## 6 Phage against infection

While improving understanding of the role of phages in controlling antimicrobial resistance (Section 2), more attention is being paid to the clinical application of phage therapy in localized and wound-associated bacterial infections.

Although the therapeutic use of phages to treat infections is not yet fully established in routine clinical practice, they have already been tested in several settings, including wound and burn infections. Bacteriophages, which are viruses that naturally infect bacteria, are now being explored as an alternative option for managing infections caused by multidrug-resistant pathogens (Islam et al., 2023b). For example, one clinical study evaluated the efficacy and tolerability of a cocktail of lytic bacteriophages targeting *Pseudomonas aeruginosa* in burn patients, comparing it with standard care (Jault et al., 2019).

Beyond these more common applications, diabetic foot ulcer (DFU) infections represent an increasingly important public health challenge. Their rising prevalence, frequent poor response to antibiotics, and growing bacterial resistance to conventional antimicrobials contribute to substantial morbidity and mortality. In this context, bacteriophages highly specific viruses that infect defined bacterial species offer a potential solution, particularly when antibiotics no longer work. Of particular interest are virulent staphylococcal phages that can lyse nearly all Staphylococcus aureus strains, including most methicillin-resistant *S. aureus* (Fish et al., 2016; Islam et al., 2025). Experimental work has shown that increasing the frequency of bacteriophage administration can enhance their effectiveness in a human ex vivo skin model, although this effect was not observed in a comparable porcine model. In both systems, however, prophylactic use of phages administering them before or at the time of bacterial exposure improved overall treatment efficacy. In addition to the PhagoBurn trial, an increasing number of compassionate-use cases, phase I safety studies and observational clinical reports have been gathered, all of which indicate that topical phage therapy is safe and could be clinically useful for certain cases of infections of wounds and diabetic ulcers. **Table 3** summarizes key clinical and translational studies supporting the use of phage therapy in burn wounds, chronic wound infections, and diabetic foot ulcers.

**Table 3:** Use of phage to treat infection.

Type	Pathogen	Purpose (Study)	Phage used	Efficacy	Citation
Burn wounds	*P. aeruginosa*	Test topical phage cocktail vs. standard care	PhagoBurn cocktail	Safe, modest efficacy	(Jault et al., 2019)
Chronic wounds	Mixed bacteria	Phase I safety study	Intralytix cocktail	Safe; tolerable	(Rhoads et al., 2009)
Diabetic ulcers	*S. aureus*	Case series (compassionate use)	Topical staph phage	Improved wound healing	(Fish et al., 2016)
Diabetic foot	*S. aureus*	Adjunct phage therapy	Staph-targeted phage	6/10 recovered	(Young et al., 2023)
Equine skin	*S. aureus*	Treat equine pyoderma	Equine phage mix	Healed lesions; safe	(Marshall and Marsella, 2023)
Burn wound model	*S. aureus*, *P. aeruginosa*	Porcine & ex vivo skin model	Specific lytic phages	Reduced bacterial load	(Molendijk et al., 2024)
Chronic wounds (review)	Various	Review of topical phage therapy	Various	Generally safe; effective in many cases	(Duplessis and Biswas, 2020)

## 7 Phage against cancer

In addition to antibacterial activity, bacteriophages have been found to be useful as vectors for targeted drug delivery, immunomodulation and cancer therapy. Conventional cancer treatment primarily relies on four major modalities: surgery, chemotherapy, radiotherapy, and hormone therapy. In recent years, however, several advanced therapeutic strategies have emerged to complement these traditional approaches. Among them, immunotherapy has gained considerable attention due to its ability to stimulate the body’s immune system to recognize and eliminate tumor cells. Current immunotherapeutic strategies include tumor-specific monoclonal antibodies, immunostimulatory cytokines, and dendritic cell-based therapies (Manivannan et al., 2022). Additional approaches involve peptide vaccines, pattern-recognition receptor agonists, immunomodulatory antibodies, and agents capable of inducing immunogenic cell death (Goracci et al., 2020). These innovations are part of a broader field of nanobiotechnology, which aims to develop novel systems for the detection and treatment of cancer (Lu and Collins, 2007). Within this context, bacteriophage-based platforms have emerged as promising tools for cancer diagnosis and therapy.

One of the most influential developments in this area is phage display technology, particularly the system based on the filamentous M13 bacteriophage, which was adapted in the late 1980s and early 1990s to generate human antibodies and identify biologically active peptides (Ragothaman and Yoo, 2023). Today, phage display is widely used to screen peptide libraries and identify molecules capable of selectively binding to tumor cells. Such tumor-targeting peptides can then be applied in targeted drug delivery or cancer imaging. Phages can also serve as vaccine platforms in two primary ways. First, they may function as DNA vaccines, delivering genetic material encoding tumor antigens into host cells. Second, they can be used as display vaccines, in which antigenic peptides are presented directly on the phage surface through phage display technology (Manivannan et al., 2022). Peptides identified through phage display often act as efficient tumor-targeting ligands and may offer advantages over traditional antibodies, including lower production costs, improved cellular uptake, and reduced immunogenicity.

**Figure 1:**
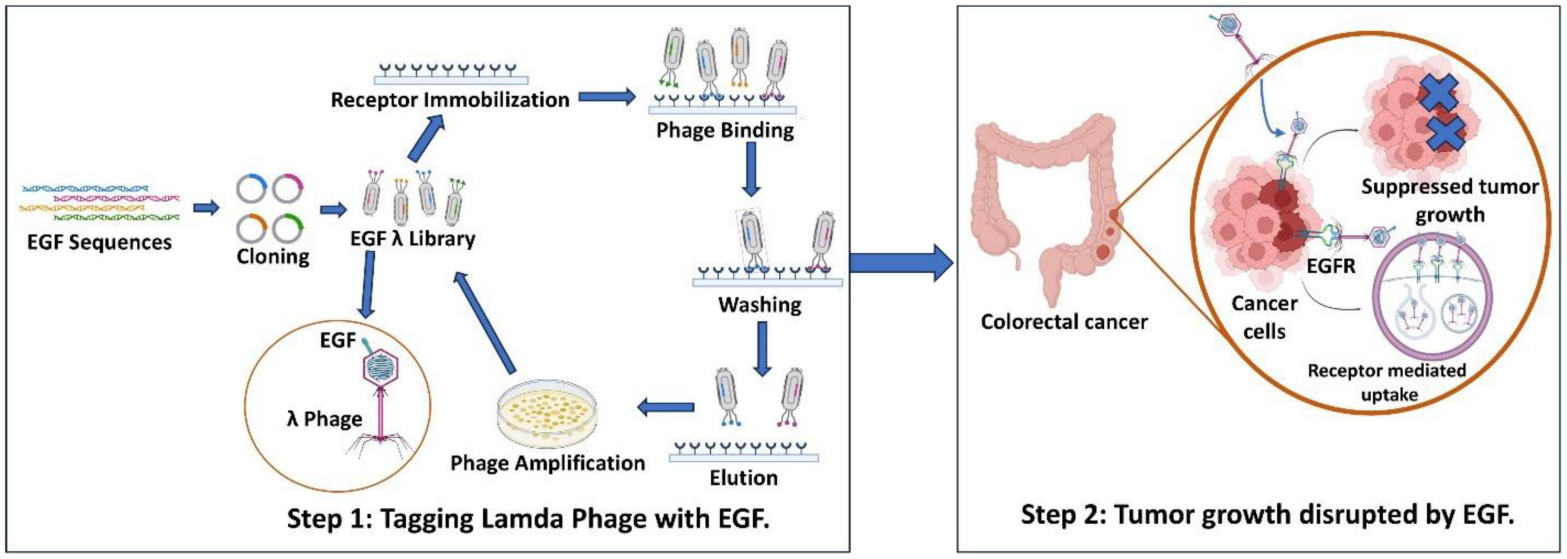
Molecular mechanism of phage display and biopanning. This schematic shows the process of genetic engineering of lambda bacteriophages that are modified with genes from peptide targets fused to the genes of the bacteriophage coat protein. This gives a direct genotype to phenotype relationship as the peptide is displayed on the viral surface, while the viral genome carries the peptide’s corresponding gene. The biopanning process is a five-step procedure that includes library construction, target exposure, stringent washing, elution, and re-amplification. This screening is a repetitive process which allows the enrichment of peptides with higher binding affinity for the tumor-associated biomarkers with higher specificity.

Beyond their role in vaccine development, phage capsids can also be repurposed as nanocarriers for targeted therapeutic delivery. For example, capsids derived from bacteriophages such as MS2 bacteriophage and P22 bacteriophage have been engineered as delivery systems. The MS2 capsid has been used to transport therapeutic non-coding RNAs into hepatocellular carcinoma cells, whereas P22 capsids have been applied to deliver enzymes such as cytochrome P450 into cervical cancer cells (Li et al., 2023). In colorectal cancer research, investigators have developed three-dimensional tumor spheroid models consisting of fibroblasts and colorectal cancer cells (**Figure 1**). These models better replicate the structural and functional interactions between tumor cells and stromal components observed *in vivo* (Huh et al., 2022). Using this system, researchers evaluated both wild-type and engineered forms of Bacteriophage Lambda, including variants displaying epidermal growth factor (EGF) on their capsids. Fluorescently labeled phages demonstrated the ability to penetrate the tumor microenvironment. Notably, the EGF-displaying phage showed enhanced and sustained uptake by tumor cells compared with the wild-type phage. Once internalized, these phages accumulated within intracellular compartments and underwent normal cellular processing.

Importantly, the λ phage itself did not significantly affect the viability or structural integrity of mature tumor spheroids. However, the engineered phage was able to inhibit the early stages of tumor formation, suggesting that phage particles may exert subtle antitumor effects even in the absence of an additional therapeutic payload (Huh et al., 2022). These findings highlight the potential of engineered bacteriophages as targeted delivery vehicles for cancer therapeutics. Despite these promising advances, several biological, pharmacological, and regulatory barriers continue to limit the clinical translation of phage-based cancer therapies. Although phage display technology has transformed the discovery of high-affinity ligands for cancer biomarkers, the successful development of phage-derived therapeutics requires overcoming a number of important challenges. The most popular platforms include M13, T7 and Lambda phages, which are engineered to present multiple copies of foreign antigens on their surface proteins (Adhya et al., 2014; Bush et al., 2026). Although they are highly specific, there is a significant clinical problem of biodistribution and a short half-life. The reticuloendothelial system recognizes most of the phages and this process can lead to a collection of the phages in the liver and spleen (Ooi and Yeh, 2024). Further, chronic exposure could result in immunogenic effects such as neutralization by antibodies and activation of innate immune pathways, which may diminish treatment effectiveness (Islam et al., 2023a). Another major impediment is the penetration of the tumor. The diffusion of phage particles into deeper regions of tumors might be hampered by the dense extracellular matrices and high interstitial pressure in the tumors (Wang et al., 2025). To overcome these disadvantages, the use of matrix-degrading enzymes and smaller fragments that are displayed on the surface of phages to facilitate tissue infiltration have been explored recently (Li et al., 2024). Moreover, the regulation of the development and production of complex phage therapeutics is difficult due to the absence of standardized manufacturing and quality control processes, and a lack of long-term data on biodistribution and persistence in humans (Sunderland et al., 2017; Islam et al., 2023a). Considering these biological, pharmacological and regulatory factors will be crucial to realize the clinical translation of phage-based precision oncology. Accumulating evidence from experimental and clinical studies indicates that phage-based therapies are generally well tolerated in humans and rarely produce significant adverse effects. In addition, bacteriophages are relatively stable biological entities and can often be stored for extended periods without substantial loss of activity (Li et al., 2020). Nevertheless, before phage-based approaches can be integrated into routine clinical practice, they must undergo rigorous experimental validation and comprehensive clinical trials. Another challenge to the broader adoption of phage therapy is the persistent misconception that bacteriophages may act as causative agents of human disease. Addressing this misunderstanding is important for both public acceptance and regulatory approval. If successfully developed and implemented, phage-based therapeutic platforms could offer substantial benefits, not only in cancer treatment but also in combating infections caused by multidrug-resistant (MDR) bacteria, which represent a major global health threat (Rehman et al., 2019).

## 8 Applications in veterinary medicine and animal husbandry

Due to the current concerns about antimicrobial resistance, the application of bacteriophages for veterinary use has been brought to the attention of veterinarians and researchers of animal production. Many studies have been performed and have shown that phages are effective against significant bacterial pathogens of livestock and poultry, such as *Salmonella enterica*, *Escherichia coli*, *Campylobacter jejuni*, and *Staphylococcus aureus*, as summarized in **Table 4** (Loponte et al., 2021; Huang et al., 2022). In poultry, the use of phages has been demonstrated to decrease bacterial colonization and disease burden, and providing phage prior to infection has been observed to be more effective in reducing bacterial load than after infection (Atterbury et al., 2007; Ahmadi et al., 2016; Nale et al., 2020). Phages have also been successfully used to disinfect the environment, and it has been shown that they can be used to reduce mortality caused by avian colibacillosis when applied via the poultry bedding systems (El-Gohary et al., 2014).

**Table 4:** Use of phage in veterinary and animal husbandry.

Animal	Used phage	Purpose	Outcome	Citation
Dairy cows	Phage cocktail against drug-resistant *E. coli*	Treat bovine mastitis caused by *E. coli*	reduced bacterial counts, somatic cells & inflammatory markers;	(Guo et al., 2021)
Lactating dairy cattle	*Staphylococcus aureus*–specific phage (intramammary infusion)	Treat subclinical *S. aureus* mastitis	Cure rate 16.7% in phage-treated quarters vs 0% in saline controls;	(Gill et al., 2006)
Broiler chickens (meta-analysis)	Multiple phage studies (*Salmonella, Campylobacter, E. coli*)	Systematic synthesis of phage efficacy in poultry	Significantly lower targeted bacterial concentrations.	(Desiree et al., 2021)
Pigs (weaned piglets)	Dietary bacteriophage supplementation (commercial mixes)	Growth promotion + diarrhoea reduction as antibiotic alternative	Decreased feed to gain ratio (F/G) & diarrhoea incidence.	(Zeng et al., 2021)
Pigs (meta-analysis)	Multiple phage intervention studies	Systematic assessment of phage therapy in pigs	Significantly reduces target bacterial concentrations in pigs.	(Desiree et al., 2021)
Swine (review & experiments)	Phages targeting *Salmonella, E. coli*, others	Improve gut health, control pathogens in swine operations	Reduced pathogen load and improve nutrient digestibility/health in pigs.	(Chen et al., 2025b)
Fish larvae/finfish	Vibriophage KVP40 & others	Prevent/treat vibriosis in marine/farmed fish larvae	Phage KVP40 reduced or delayed larval mortality.	(Silva et al., 2014)
Shrimp (*Litopenaeus vannamei*)	Nucleus-forming vibriophage cocktail	Treat Vibrio infections in shrimp aquaculture	Reduced Vibrio burden & improved shrimp survival (~91% survival).	(Thammatinna et al., 2023)
Shrimp aquaculture	Phage cocktails prepared for *Vibrio* spp.	Prophylactic/therapeutic control of vibriosis	Reduced Vibrio counts and increased survival vs untreated controls.	(Chen et al., 2019)
Shrimp farms/aquaculture effluents	Novel vibriophages isolated from aquaculture	Isolate phages suited to local aquaculture pathogens	Isolated phages active against *Vibrio harveyi* & *V. parahaemolyticus*	(Moon et al., 2025)
Aquaculture (fish/shrimp)	Two virulent vibriophages (characterization)	Evaluate potential for translation into aquaculture phage therapy	Characterised phages showed lytic activity and *in vivo* potential.	(Shi et al., 2026)

In addition to poultry, phage has been successfully applied in cattle and swine production. Phages against *Staphylococcus* spp. have been studied for prevention and treatment of bovine mastitis and phage preparations have been added to the feed of pigs to combat Salmonella colonization and enhance animal health (O’Flaherty et al., 2005; Thanki et al., 2022). The results suggest that phage therapy has the potential to significantly decrease the use of antibiotics and the spread of food borne pathogens along the food chain. In addition, recent genomic mining efforts have further broadened the selection of candidate phages to be used in agriculture, most notably for Salmonella infections (Gendre et al., 2022).

Phage therapy also has shown to be effective in companion animals, though less studied. Initial studies have shown good results in dogs infected with *Pseudomonas aeruginosa* associated with otitis and *Staphylococcus intermedius* related to pyoderma (Hawkins et al., 2010; Damborg et al., 2016; Silva et al., 2021). Phages have also been considered as promising biocontrol agents in aquaculture, including in fish and shrimp production, where mortality rates have been reduced and fish and shrimp survival increased, for bacterial pathogens like *Vibrio*, *Pseudomonas* and *Aeromonas* species (Park and Nakai, 2003; Richards, 2014; Svircev et al., 2018; Huang et al., 2022). Yet, although some positive outcomes have been seen, there are limitations. In many studies dealing with veterinary and aquaculture research, experiments are carried out in an uncontrolled environment, with limited numbers of samples, or with no standardized treatment methods. Reporting is inconsistent among studies on dosage, administration and treatment schedules in aquaculture, which makes it difficult to compare studies. In addition, the impacts of widespread phages release into aquatic environments have not yet been well studied (Richards, 2014). Future research should also consider the ecological implications of releasing therapeutic phages into marine environments (Meaden and Koskella, 2013). Overall, the available data suggests that phage therapy could be a viable means of maintaining bacterial diseases in animal production systems. However, extensive field testing, standardized efficacy testing and more accurate regulation will be necessary to implement phage-based interventions on a larger scale in the veterinary and aquaculture industries.

## 9 Phage in biofilm eradication

Researchers have proposed a strategy in which highly efficient, biofilm-specific EPS-degrading enzymes are engineered into bacteriophages that already kill bacteria in a species-specific manner (Lu and Collins, 2007). Instead of searching the environment for rare phages with natural biofilm-dispersing activity, this approach would allow the design of a broad library of phages tailored to dismantle particular biofilms. Once these engineered phages infect bacteria within a biofilm, they hijack the host’s machinery and replicate, generating high local concentrations of both lytic phage particles and biofilm-degrading enzymes, even when starting from relatively low initial doses. Because phage replication and bacterial lysis proceed rapidly, this dual attack direct bacterial killing plus enzymatic matrix disruption could provide an efficient, self-amplifying method to clear biofilms in environmental, industrial, and clinical settings.

A key advantage of this design is that it eliminates the need to produce and deliver large quantities of purified enzymes to sites of infection that may be difficult to reach. Instead, the phages themselves act as targeted factories and delivery vehicles, which should enhance the ability of phage therapy to eradicate biofilm-associated infections. Ongoing advances in genome sequencing and synthetic biology such as phage genome refactoring and large-scale DNA synthesis (Chan et al., 2005; Itaya et al., 2005; Bumunang et al., 2023) are expected to further accelerate the development of these engineered enzymatic phages and expand beyond the relatively small set of naturally occurring biofilm-degrading phages that have been isolated so far. As bacteriophage therapy becomes better understood and more widely adopted, phages equipped with biofilm-degrading enzymes could emerge as practical tools for controlling biofilms in a wide range of settings (Lu and Collins, 2007). Several experimental studies already illustrate the clinical potential of such approaches. For example, bacteriophage MA-1, isolated from wastewater, has been used to target biofilms formed by multidrug-resistant (MDR) Pseudomonas aeruginosa (Adnan et al., 2020). Treatment with MA-1 reduced the growth rate of P. aeruginosa and significantly diminished biofilm biomass within 6 hours. Notably, the study highlighted that this phage can enzymatically degrade alginate polymers, enabling it to disrupt even long-standing biofilms, including those that had been allowed to mature for 20 days. Phages can also compromise biofilms indirectly by killing bacteria before they attach to surfaces or after they have already colonized them (Adnan et al., 2020). In another investigation, the activity of PB1-like, phiKZ-like, and LUZ24-like phages against MDR P. aeruginosa was evaluated under various growth conditions. Each phage alone was capable of suppressing both planktonic and biofilm forms of the pathogen. PhiKZ-like phages were particularly effective against planktonic cells, whereas LUZ24-like phages showed the greatest ability to disrupt biofilms formed by antibiotic-resistant isolates. Furthermore, a phage cocktail containing all three types displayed superior activity compared with any single phage alone, underscoring the potential benefits of combining complementary phages to enhance biofilm eradication (Latz et al., 2017). **Table 5** summarizes key studies on the use of bacteriophages against microbial biofilms.

**Table 5:** Use of phage against microbial biofilm.

Purpose	Pathogen/target	Reported efficacy/organism	Outcome	Citation
Dispersing biofilms with engineered enzymatic bacteriophage	to degrade biofilm matrix while killing cells.	*E. coli* biofilms (model system)	Engineered T7-DspB dramatically increased biofilm removal vs wild-type phage.	(Lu and Collins, 2007)
Bacteriophage-Derived Depolymerases against Bacterial Biofilms	depolymerases as antibiofilm agents and their translational potential.	Broad - *Klebsiella, Pseudomonas, E. coli, Salmonella*	producing 1–4 log reductions or improved antibiotic susceptibility in combination.	(Soontarach et al., 2024)
Phage therapy against *Pseudomonas aeruginosa*	*P. aeruginosa* biofilms with phage or phage cocktails.	*Pseudomonas aeruginosa* biofilms	reduced biofilm biomass, cell counts and improved outcomes in animal/*in vitro* models	(Fong et al., 2017; Maffei et al., 2024; Soontarach et al., 2024)
Bacteriophage-derived depolymerases into therapeutics.	phage depolymerases as antibiofilm agents.	Broad -(polysaccharide matrices across species)	Preclinical data are promising	(Eghbalpoor et al., 2024)
Bacteriophage-mediated biofilm control	Phage enzymes, combos and biofilm-specific strategies.	Multiple species/settings (medical, industrial)	Reduced biofilms but emphasize variability and resistance/tolerance observed in mature biofilms.	(Ponce Benavente et al., 2024)
Improvement of phage biofilm activity	improved anti-biofilm activity and reduced resistance tradeoffs.	*Pseudomonas* biofilms (model)	improved phage performance against biofilm challenges.	(Kunisch et al., 2024)
Bacteriophage against Staphylococcus aureus in biofilm assays & models.	lytic phage in staphylococcal biofilms	*Staphylococcus aureus* (biofilms)	Phage penetrated biofilms and interacted with cells;	(González et al., 2018)
Phage depolymerase for treatment of biofilm infections.	Depolymerases as antibiofilm therapeutics.	Broad - (biofilm EPS targets)	Improved antibiotic penetration and reduce biofilm burden in models;	(Latka and Drulis-Kawa, 2020)
Phages against bacterial biofilms (pathogenic)	phage enzymes, combined therapies and pharma considerations.	Multiple pathogens (*Pseudomonas*, Staph, Klebsiella, etc.)	Phage depolymerases reduce biomass and improve antibiotic efficacy.	(Zurabov et al., 2023)
*Vibriophage KVP40 against reduction of larval mortality*	Application of vibriophage to fish/shrimp larvae	*Vibrio anguillarum* (aquaculture)	KVP40 reduced or delayed larval mortality from *Vibrio* infection.	(Silva et al., 2014)
Bacteriophage activity against *S. aureus* biofilms	Evolve phages on biofilms to select variants with improved anti-biofilm activity.	*Staphylococcus aureus* biofilms	Produced phage variants with enhanced activity compared with parental phages.	(Ponce Benavente et al., 2024)

## 10 Limitations and future perspectives

Despite the remarkable progress in phage-based therapeutics, several critical limitations must be addressed before these approaches can be integrated into mainstream clinical practice. The narrow host range of most bacteriophages remains a fundamental constraint, necessitating personalized approaches that are logistically challenging and difficult to scale. Current phage susceptibility testing methods lack standardization across laboratories, and their correlation with clinical outcomes has not been rigorously validated through prospective trials. The pharmacokinetic behavior of phages in humans remains poorly characterized, with limited understanding of their distribution, clearance, and immunogenicity following various routes of administration. Regulatory pathways for phage products are fragmented globally, with uncertainty regarding whether phages should be classified as drugs, biologics, or living medicines, creating barriers to commercialization and widespread adoption.

The emergence of phage resistance represents an unavoidable evolutionary outcome that must be prospectively managed through rationally designed cocktails and combination strategies. While phage-antibiotic synergy offers therapeutic advantages, the mechanisms underlying these interactions are incompletely understood and may vary unpredictably across different pathogen-drug-phage combinations. The paradoxical observation that phage predation can occasionally accelerate plasmid-mediated resistance transfer under specific conditions warrants further investigation to identify risk factors and develop mitigation strategies. Manufacturing and quality control pose substantial challenges, particularly for personalized phage preparations that require rapid turnaround while maintaining stringent purity and potency standards.

Future directions should prioritize the development of well-characterized, off-the-shelf phage cocktails with broad coverage against prevalent clinical pathogens, potentially through the creation of phage banks or libraries with defined genetic and functional properties. Advances in synthetic biology offer opportunities to engineer phages with enhanced properties, including expanded host range, biofilm-degrading enzymes, CRISPR-Cas antimicrobial payloads, and reduced immunogenicity. Simultaneously, the application of machine learning to phage discovery and prediction of phage host has evolved from an empirical to a more data-driven approach. Machine learning models like CHERRY (Boeckaerts et al., 2024; Shang et al., 2025) use multimodal data, such as genome similarity and CRISPR-derived signals, in a graph-based deep learning model to predict phage-host interaction at the species level with false discovery rates less than 10%. Similarly, VirHostMatcher-Net is able to boost prediction accuracy for host prediction by fusing alignment-free genomic similarity measures and co-abundance network features, with prediction accuracy significantly increased compared to other methods (Nami et al., 2021). Integrated platforms like VIBRANT and VirSorter2 can now automatically recover viruses, annotate them, and assess their metabolic capabilities from complex metagenomic samples (Liu et al., 2024). Deep-learning based tools such as Seeker can be used to identify previously unknown phages that show limited sequence similarity to known viral families. Though these advances have been made, predicting phage specificity at the strain level is still a challenge and a critical requirement for precision phage therapy. New methods are increasingly using protein language models trained on massive protein sequence collections to model the evolutionary and functional properties of viral proteins, to allow host prediction at subspecies level (Briers et al., 2023). Establishment of standardized computational benchmarks alongside comprehensive, well-designed, and suitably powered randomized controlled trials will be crucial to validate these technologies and bring phage therapy into the realm of clinical evidence. Standardizing susceptibility testing methods, pharmacokinetic-pharmacodynamic modeling, and validated biomarkers of treatment response will more successfully aid the clinical translation of phage therapeutics. These challenges will need to be met by a multidisciplinary approach between microbiologists, clinicians, bioinformaticians, regulators and industry partners that is crucial to the realization of the therapeutic potential for bacteriophages over the next decade.

## 11 Conclusion

Bacteriophages have seen significant research in recent years, alongside the global problem of antimicrobial resistance, and with the progress of synthetic biology, genomics, and precision medicine. In addition to their classical bacteriolytic properties, phages have shown to have other therapeutic opportunities such as disrupting biofilms, restoring antibiotic susceptibility and delivering antimicrobial or genetic payloads to specific targets. Their applicability as new biomedical tools is further illustrated by their use in agriculture, Veterinary medicine, and cancer research. Although these are promising advances, there are still difficulties with implementing phage therapy more widely into routine clinical use. While there are encouraging results reported in compassionate use cases, observational studies and early clinical investigations, there is a dearth of large-scale and well-controlled randomized clinical trials. For this reason, well established evidence for efficacy for a variety of clinical indications is still not available. Furthermore, there remain a number of key challenges to address, such as phage pharmacokinetics, host immune responses, optimal dosing regimens, standardized susceptibility testing, manufacturing consistency, and regulatory approval pathways. Phage resistance and the variability in phage–antibiotic interactions also pose challenges for clinical application. In the future, rapid progress in machine learning, synthetic biology and genome engineering could speed up the process of discovering phages, enhance the host prediction, and help create new generation therapeutic phages. The future of phage therapy in clinical practice, however, will require the systematic validation in clinical trials, harmonized regulations and ongoing multi-disciplinary research and cooperation. Bacteriophages are an exciting addition to the arsenal of traditional antimicrobial interventions; however, the potential of these interventions will be realized only when these significant scientific, translational, and regulatory hurdles are overcome.
